# The association of maternal prenatal psychosocial stress with vascular function in the child at age 10–11 years: findings from the Avon longitudinal study of parents and children

**DOI:** 10.1177/2047487313486039

**Published:** 2013-04-04

**Authors:** Aimée E van Dijk, Karen Dawe, John Deanfield, Karien Stronks, Reinoud JBJ Gemke, Tanja GM Vrijkotte, Debbie A Lawlor

**Affiliations:** 1 University of Amsterdam, Amsterdam, The Netherlands; 2 Public Health Service of Amsterdam (GGD), Amsterdam, The Netherlands; 3 University of Bristol, Bristol, UK; 4 University College London, London, UK; 5 VU University Medical Center, Amsterdam, The Netherlands

**Keywords:** Arterial stiffness, blood pressure, child, developmental origins of health and disease, fetal development, pregnancy, psychological, stress

## Abstract

**Objective:**

To investigate whether (1) maternal psychosocial stress (depression/anxiety) during pregnancy is associated with offspring vascular function and (2) whether any association differs depending on the gestational timing of exposure to stress. We also investigated whether any association is likely to be due to intrauterine mechanisms by (3) comparing with the association of paternal stress with offspring vascular function and (4) examining whether any prenatal association is explained by maternal postnatal stress.

**Methods and results:**

Associations were examined in a UK birth cohort, with offspring outcomes (systolic and diastolic blood pressure, SBP and DBP, endothelial function assessed by brachial artery flow-mediated dilatation (FMD); arterial stiffness assessed by carotid to radial pulse wave velocity (PWV), brachial artery distensibility (DC), and brachial artery diameter (BD) assessed at age 10–11 years (*n* = 4318). Maternal depressive symptoms and anxiety were assessed at 18 and 32 weeks gestation and 8 months postnatally. Paternal symptoms were assessed at week 19. With the exception of DBP and BD, there were no associations of maternal depressive symptoms with any of the vascular outcomes. Maternal depressive and anxiety symptoms were associated with lower offspring DBP and wider BD, though the latter attenuated to the null with adjustment for confounding factors. Paternal symptoms were not associated with offspring outcomes. Maternal postnatal depressive symptoms were associated with lower offspring SBP.

**Conclusions:**

We found no evidence to support the hypothesis that maternal stress during pregnancy adversely affects offspring vascular function at age 10–12 years via intrauterine mechanisms.

## Introduction

Maternal stress or the administration of glucocorticoids (‘stress hormones’) have been linked to altered fetal growth and might have permanent effects on the tissue structure and function of the offspring.^1^ For example, the hypothalamic–pituitary–adrenal axis, which is a key part of the neuroendocrine stress response, and the renin–angiotensin system may be affected by intrauterine exposure to maternal stress, and this exposure may lead to adult risk of cardiovascular disease.^2–17^

Convincing evidence for the effects of fetal programming on cardiovascular risk factors comes from animal studies, in which prenatal exposure to excess glucocorticoids results in persistent elevation of arterial blood pressure (BP) in adulthood, with potential sex differences (for review, see Drake et al.^18^). There is also some evidence from human studies. For example, antenatal glucocorticoid administration is linked with high BP in the fetus^19^ and in adolescence.^20^ Those studies have, however, been complicated by high doses of glucocorticoids, which are not comparable to levels normally occurring during pregnancy. In addition, glucocorticoid treatment usually takes place when pre-term birth is expected, adding bias because of the complications of premature birth. Few pregnant women will be treated with glucocorticoids, but the results of these studies have been used to suggest that common mental complaints (depression and anxiety) associated with stress, if experienced by the mother during pregnancy might increase her offspring's risk of cardiovascular disease. If this is true it would have important public health implications, but to our knowledge few studies have examined this.

In a previous study in a Dutch population-based birth cohort (ABCD study), the presence of multiple psychosocial stressors during early pregnancy was associated with higher BP in the offspring at age five.^21^ However, we are unaware of any other human studies of this association and this needs further replication and additional exploration to see if any association is likely to reflect a causal intrauterine mechanism.

The aim of this study was to examine whether (1) maternal psychosocial stress (depression/anxiety) during pregnancy is associated with offspring vascular function (assessed by systolic blood pressure (SBP), diastolic blood pressure (DBP), endothelial function assessed by brachial artery flow-mediated dilatation (FMD), arterial stiffness assessed by carotid to radial pulse wave velocity (PWV), brachial distensibility coefficient (DC), and brachial artery diameter) and (2) whether any association differs depending on the gestational timing of exposure to stress. We further examined whether there is evidence that any association of maternal stress with offspring vascular function is likely to be due to intrauterine mechanisms by comparing the associations of maternal stress with offspring outcomes to those of paternal antenatal stress with the same outcomes and by examining whether any maternal prenatal association was explained by the persistence of symptoms into the postnatal period and exposure postnatally (rather than intrauterine driving associations). The paternal comparison is an important form of negative control for exploring a causal association in observational epidemiology.^22,23^ The rationale is that if maternal stress is truly causing offspring outcomes via intrauterine mechanisms (as opposed to family based confounding) then one would expect a stronger maternal association, compared to paternal association. Examining whether any maternal pregnancy stress association with offspring outcomes is largely mediated by postnatal stress is also important as if the effect is largely due to postnatal impact then any intervention to prevent adverse offspring outcomes would want to focus on minimizing postnatal stress.

## Methods

### Study population

**Figure 1.**
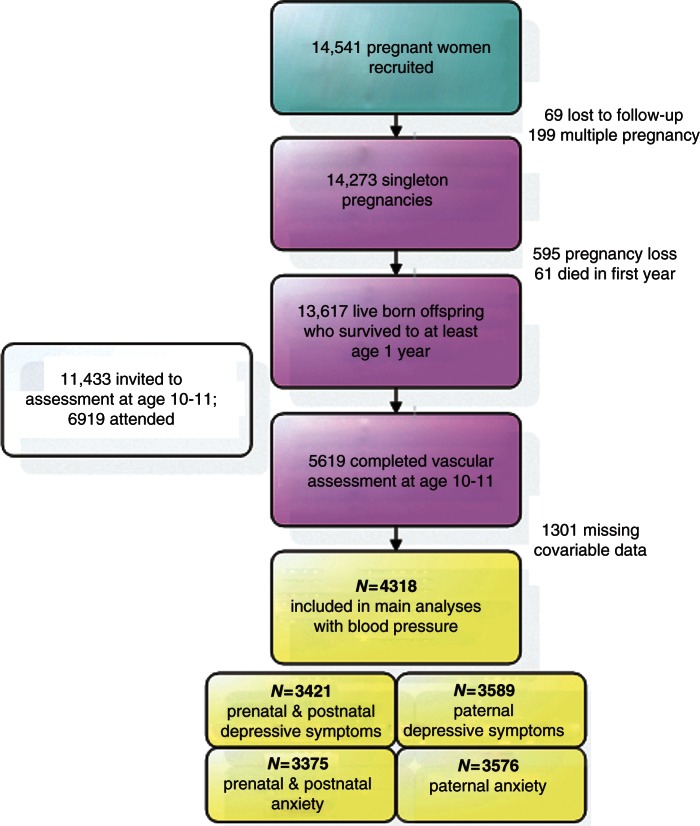
Participant flow. Data in the yellow boxes are for mother(father)–child pairs with complete data.

Data from the Avon Longitudinal Study of Parents and Children (ALSPAC), a prospective population-based birth cohort that recruited 14,541 pregnant women resident in Avon, UK with expected dates of delivery 1 April 1991 to 31 December 1992 were used. Additional details of the study can be found on the study website (www.alspac.bris.ac.uk) and in previous publications.^24–26^ Figure 1 shows the flow of participants through the study. In this study, we included mother–offspring pairs where there was a singleton pregnancy resulting in a live birth with the child surviving to at least 1 year of age. The eligible cohort for the current analyses was the 4318 mother–offspring pairs who had complete data on maternal depressive symptoms and anxiety at both gestational time points, offspring blood pressure (obtained at the 10–11-year follow-up clinic), and complete data on all covariates.

### Parental assessment of psychosocial stress

Data on depressive symptoms and anxiety were collected through postal questionnaires, which the mothers in the current sample filled out at a median gestational age of 18 weeks (mean 19; interquartile range 18–20) and median 32 weeks (mean 32; interquartile range 32–33) antenatally and median 8 months postnatally (mean 8.6; interquartile range 8.1–9.1). Fathers completed one questionnaire at a median 19 weeks of their partners’ pregnancy (mean 20; interquartile range 19–21). Maternal and paternal depressive symptoms at all time points were assessed using the Edinburgh Postnatal Depression Scale (EPDS), a widely used 10-item self-report questionnaire that has been shown to be valid in and outside the postnatal period.^27^ A pilot study of a random sample of 54 pregnant women attending a routine check-up indicated substantial overlap between the EPDS and the Center for Epidemiological Studies Depression Scale (*r*^2 ^= 0.87, *p* < 0.001). Internal consistency (Cronbach's alpha) of the EPDS exceeded 0.80 at each of the assessments.^28^

Maternal and paternal anxiety at all time points was measured using the anxiety items from the Crown–Crisp experiential index (CCEI), a validated self-rating inventory (for example, see Crisp et al.^29^); example items include ‘worry a lot’ and ‘feeling strung up inside’). In a pilot study of a random sample of 54 pregnant women attending a routine check-up, Crown–Crisp index correlated 0.70 and 0.76 with the State and Trait (respectively) subscales of the Spielberger State-trait Anxiety Inventory. Internal consistency (Cronbach's alpha) exceeded 0.80 at each of the assessments.^28^

To compare prenatal and postnatal maternal stress we categorized all EPDS and CCEI scores into ‘high levels of anxiety/depressive symptoms’ (yes versus no) by dichotomizing at the 80th percentile (those classified as having ‘high levels’ included those with values at or higher than the 80th percentile; those classified as ‘normal’ included those with values lower than the 80th percentile). We then further categorized participants into those with normal levels at all time points; prenatal high levels only; postnatal high levels only, or pre- and postnatal high levels.

### Vascular function in the child

Vascular phenotypes were assessed at the 10–11-year follow-up assessment and detailed descriptions of the measurements have been previously reported.^30,31^ Blood pressure was measured with the automatic oscillometric method. For PWV, pressure pulse waveforms were recorded transcutaneously using a high-fidelity micromanometer (SPC-301; Millar Instruments, Houston, TX, USA) from the radial and carotid pulse, synchronous with the ECG signal. Integral software processed the data to calculate the mean time difference between R-wave and pressure wave on a beat-to-beat basis over 10 s, and the PWV was then calculated using the mean time difference and arterial path length between the two recording points (SphygmoCorversion 7.1; Scanmed, UK). Ultrasound images of the right brachial artery were used to measure FMD and DC. Images were recorded onto SVHS video using an ALOKA 5500 high-resolution ultrasound system with a 5–10 MHz linear array probe (Keymed, UK) and the measurements were undertaken later from the videos at the Vascular Physiology Unit, Institute of Child Health, London. The right brachial artery was imaged 5–10 cm above the antecubital fossa. FMD was induced by a 5-min inflation of a pneumatic cuff, placed around the forearm immediately below the medial epicondyle, to 200 mmHg followed by rapid deflation using an automatic air regulator. The diameter of the brachial artery was measured using edge detection software (Brachial Tools, MIA, IA, USA) from ECG-triggered ultrasound images captured at 3 s intervals throughout the 11-min recording protocol. FMD was expressed as an absolute value in our analyses.^31^ The distension of the artery was determined by measuring the luminal diameter excursion from diastole to systole. Distensibility coefficient was calculated from the distension and the pulse pressure and was expressed as mean per cent change in cross-sectional area per unit change in blood pressure.

### Covariates

Maternal age, gestational age, birthweight, and offspring sex were obtained from obstetric records. Self-reported weight and height at the time of recruitment were used to calculate maternal pre-pregnancy body mass index (BMI; Pearson's correlation with measured weight at first antenatal clinic *r*^2 ^= 0.95, *p* < 0.0001). Parity was obtained from a maternal completed questionnaire at 18 weeks gestation and for these analyses we dichotomized this (any previous pregnancies yes/no). Based on questionnaire responses, the highest parental occupation was used to allocate the children to head of household occupational social class groups (classes I (professional/managerial) to V (unskilled manual workers), using the 1991 British Office of Population and Census Statistics classification). Ethnicity was based on the mothers’ self-report (White; Black Caribbean; Black African; Other Black; Indian; Pakistani; Bangladeshi; Chinese; Other). Mothers were repeatedly asked about their smoking throughout pregnancy and these data were used to generate a categorical variable (never smoked; smoked before pregnancy or in the first trimester and then stopped; smoked throughout pregnancy). A categorical variable on alcohol consumption was defined based on a question in the questionnaire at gestational week 18 (no drinking; less than 1 glass per week; more than 1 glass per week; more than 1 glass per day).

Age of the child at the time of vascular function assessment was recorded in months as they arrived at the assessment clinics. Weight was measured to the nearest 0.1 kg using Tanita scales. Height was measured to the nearest 0.1 cm using a Harpenden stadiometer.

### Statistics

Associations between the stress measures (at all time points) and offspring vascular outcome measures were explored using multivariable linear regression with the statistical package SPSS version 18.0 (SPSS, Chicago, USA). In model 1 we adjusted for sex, height, and age of the child at the time of vascular assessment. In model 2 we additionally adjusted for maternal confounders (age, ethnicity, pre-pregnancy BMI, nulliparity, social class, smoking, and alcohol consumption). In model 3 we adjusted for potential mediation of any association by gestational age at birth and birthweight, and in model 4 for potential mediation by BMI of the child at the time of vascular assessment. We explored whether there were any differences in associations by sex of the child by testing for an interaction between sex and the exposure (depressive symptoms or anxiety) in model 2, using the Wald test. The equality of betas of a psychosocial measure at two gestational time points, or between mothers and fathers, was tested using the Wald-statistic. Associations are also checked for linearity using restricted cubic splines within ordinary least squares linear regression analysis (OLS) in R (R Foundation for Statistical Computing).

## Results

Table 1 shows the characteristics of the mothers and children included in this study. Table 2 shows the correlations between the maternal depressive symptoms and anxiety scores at all three time points and the fathers’ equivalent measurements. There were strong correlations between depressive symptoms and anxiety at each time point in the mothers, and also in the fathers. Over time, in the mothers, these measurements correlated strongly with each other. There were weak to modest correlations between maternal depressive symptoms and anxiety and paternal equivalent outcomes.

**Table 1.** Participant characteristics

Study population (*n* = 4318)
Maternal
Age at delivery (years)	29.3 ± 4.4 (26.3–32.3)
White ethnicity (yes)	98.7
Pre-pregnancy body mass index (kg/m^2^)	22.8 ± 3.6 (21.0–24.7)
Obesity (body mass index ≥30 kg/m^2^) (yes)	4.5
Social class
I (professional/managerial)	7.1
II	35.9
III (non-manual)	41.7
III (manual)	6.3
IV	7.6
V (unskilled manual workers)	1.4
Primiparous (yes)	49.4
Smoking
Non-smoking	77.3
Stopped before pregnancy or in first trimester	11.1
Smoked during pregnancy	11.6
Alcohol
None	43.7
<1 glass/week	41.0
>1 glass/week	13.7
>1 glass/day	1.5
Pregnancy-related hypertension (yes)	15.5
Maternal stress
Anxiety at gestational week 18 as a continuous score	5 ± 3 (3–7)
Anxiety at gestational week 32 as a continuous score	5 ± 3 (2–7)
Depressive symptoms at gestational week 18 as a continuous score	6 ± 5 (3–9)
Depressive symptoms at gestational week 32 as a continuous score	6 ± 5 (3–10)
Pre- and postnatal anxiety categories (*n* = 3375)^a^
Low anxiety at all time points	59.9
Prenatal anxiety only	19.8
Postnatal anxiety only	5.8
High anxiety at all time points	14.5
Pre- and postnatal depressive symptom categories (*n* = 3421)^a^
Low depressive symptoms at all time points	61.6
Prenatal depressive symptoms only	15.0
Postnatal depressive symptoms only	8.7
High depressive symptoms at all time points	14.6
Child at birth
Born through caesarean section (yes)	9.6
Sex (boys)	49.4
Gestational age (weeks)	39.6 ± 1.6 (38.6–40.6)
Premature (<37 weeks) (yes)	3.7
Birthweight (g)	3456 ± 494 (1366–5546)
Child at age 10 assessment
Age (years)	10.7 ± 0.2 (10.5–10.8)
Height (cm)	144 ± 7 (140–149)
Body mass index (kg/m^2^)	18.1 ± 3.0 (16.3–20.0)
Systolic blood pressure (mmHg)	104 ± 9 (98–109)
Diastolic blood pressure (mmHg)	60 ± 8 (54–65)
Flow-mediated dilatation (mm) (*n* = 3830)	0.21 ± 0.08 (0.15–0.27)
Pulse wave velocity (m/s) (*n* = 4286)	7.55 ± 1.22 (6.75–8.35)
Distensibility coefficient (% per mmHg) (*n* = 3943)	12.5 ± 6.1 (9.0–16.0)
Brachial artery diameter (mm) (*n* = 3830)	2.67 ± 0.30 (2.47–2.86)

Values are mean ± SD (IQR) or %

a Low anxiety/depressive symptoms both pre- and postnatally: all scores for the respective exposure (anxiety or depressive symptoms) lower than the 80th percentile. Prenatal anxiety/depressive symptoms: one or both of the prenatal scores at or higher than the 80th percentile, but the postnatal score lower than the 80th percentile. Postnatal anxiety/depressive symptoms: both prenatal scores lower than the 80th percentile and the postnatal score at or higher than the 80th percentile. High anxiety/depressive symptoms: all scores at or higher than the 80^th^ percentile. 80^th^ percentile scores: Anxiety: 7 at gestational week 18; 7 at gestational week 32; 6 at baby age 8 months. Depressive symptoms: 10; 11; 8 respectively.

**Table 2.** Correlation coefficients between maternal depressive symptoms and anxiety at gestational weeks 18 and 32, and at 8 months postnatally, and paternal depressive symptoms and anxiety at partner's gestational week 19

	18 weeks		32 weeks		Postnatal		Paternal	
	EPDS	CCEI	EPDS	CCEI	EPDS	CCEI	EPDS	CCEI
EPDS depressive symptoms at week 18	1
*n* = 4318
CCEI anxiety at week 18	0.74	1
*n* = 4318	*n* = 4318
EPDS depressive symptoms at week 32	0.64	0.57	1
*n* = 4318	*n* = 4318	*n* = 4318
CCEI anxiety at week 32	0.60	0.67	0.76	1
*n* = 4318	*n* = 4318	*n* = 4318	*n* = 4318
EPDS depressive symptoms 8 months postnatal	0.50	0.46	0.54	0.50	1
*n* = 4173	*n* = 4173	*n* = 4173	*n* = 4173	*n* = 4173
CCEI anxiety 8 months postnatal	0.47	0.53	0.49	0.56	0.76	1
*n* = 4181	*n* = 4181	*n* = 4181	*n* = 4181	*n* = 4142	*n* = 4181
Paternal EPDS depressive symptoms	0.24	0.19	0.20	0.18	0.17	0.16	1
*n* = 3589	*n* = 3589	*n* = 3589	*n* = 3589	*n* = 3475	*n* = 3484	*n* = 3589
Paternal CCEI anxiety	0.15	0.14	0.13	0.11	0.12	0.12	0.68	1
*n* = 3576	*n* = 3576	*n* = 3576	*n* = 3576	*n* = 3463	*n* = 3473	*n* = 3545	*n* = 3576

All *p*-values are <0.001

CCEI, Crown–Crisp experiential index; EPDS, Edinburgh Postnatal Depression Scale.

There was no strong statistical evidence of interaction by sex and therefore all multivariable association results are presented with both females and males combined. Furthermore, there was no evidence for non-linear associations: we therefore present results examining linear associations.

At both gestational ages, there was no strong evidence that maternal depressive symptoms or anxiety were associated with adverse cardiovascular function in any of the multivariable models (Table 3). Maternal depressive symptoms in pregnancy at both time points were associated with larger brachial artery diameter in the confounder-adjusted model (model 2), but with some evidence that these associations might be mediated by birthweight/gestational age and offspring adiposity at the time of assessment of vascular function (models 3 and 4). Depressive symptoms at 18 weeks gestation were also associated with lower diastolic blood pressure. Maternal anxiety at 18 weeks gestation was also associated with brachial artery diameter (model 2), again with some evidence of mediation by birthweight/gestational age and offspring adiposity.

**Table 3.** Multivariable associations of maternal depressive symptoms and anxiety at two gestational time points with offspring vascular phenotypes at mean age 10.7 years (*n* = 4318)

	Systolic blood pressure			Diastolic blood pressure			Flow-mediated dilatation			Pulse wave velocity			Distensibility coefficient			Brachial artery diameter		
	β	95% CI		β	95% CI		β	95% CI		β	95% CI		β	95% CI		β	95% CI	
		Lower	Upper		Lower	Upper		Lower	Upper		Lower	Upper		Lower	Upper		Lower	Upper
Depressive symptoms
Gestational week 18
Model 1	−0.038	−0.096	0.021	−0.074*	−0.126	−0.022	0.000	−0.001	0.001	−0.004	−0.012	0.004	−0.011	−0.053	0.031	0.003*	0.001	0.005
Model 2	−0.051	−0.111	0.008	−0.083*	−0.136	−0.03	0.000	−0.001	0.001	−0.005	−0.013	0.004	−0.014	−0.057	0.029	0.002*	0.000	0.004
Model 3	−0.051	−0.110	0.009	−0.082*	−0.135	−0.029	0.000	−0.001	0.001	−0.005	−0.013	0.004	−0.014	−0.057	0.029	0.002*	0.000	0.004
Model 4	−0.053	−0.112	0.006	−0.084*	−0.136	−0.031	0.000	−0.001	0.000	−0.004	−0.012	0.004	−0.014	−0.058	0.029	0.002*	0.000	0.003
Gestational week 32
Model 1	−0.039	−0.095	0.016	−0.022	−0.072	0.027	0.000	0.000	0.001	−0.002	−0.010	0.006	0.012	−0.028	0.051	0.003*	0.001	0.004
Model 2	−0.052	−0.108	0.004	−0.029	−0.08	0.021	0.000	0.000	0.001	−0.003	−0.010	0.005	0.009	−0.032	0.050	0.002*	0.000	0.004
Model 3	−0.052	−0.108	0.004	−0.028	−0.078	0.022	0.000	0.000	0.001	−0.002	−0.010	0.005	0.009	−0.032	0.049	0.002	0.000	0.004
Model 4	−0.058	−0.113	−0.002	−0.032	−0.082	0.018	0.000	0.000	0.001	−0.001	−0.009	0.006	0.009	−0.032	0.049	0.001	−0.001	0.003
Wald‐statistic (model 1)	*p* = 0.89	*p* = 0.02	*p* = 0.29	*p* = 0.67	*p* = 0.20	*p* = 0.94
Anxiety
Gestational week 18
Model 1	−0.029	−0.108	0.049	−0.034	−0.104	0.036	0.000	−0.001	0.000	−0.002	−0.013	0.009	0.014	−0.043	0.070	0.004*	0.001	0.006
Model 2	−0.046	−0.126	0.034	−0.043	−0.114	0.028	−0.001	−0.001	0.000	−0.003	−0.014	0.008	0.013	−0.045	0.070	0.003*	0.000	0.006
Model 3	−0.044	−0.123	0.036	−0.041	−0.112	0.030	−0.001	−0.001	0.000	−0.003	−0.014	0.008	0.013	−0.045	0.070	0.003*	0.000	0.005
Model 4	−0.048	−0.127	0.031	−0.044	−0.115	0.027	−0.001	−0.001	0.000	−0.003	−0.013	0.008	0.012	−0.045	0.070	0.002	0.000	0.005
Gestational week 32
Model 1	−0.018	−0.095	0.059	−0.040	−0.109	0.028	0.000	0.000	0.001	0.003	−0.008	0.013	0.024	−0.032	0.080	0.003*	0.000	0.005
Model 2	−0.034	−0.112	0.044	−0.048	−0.118	0.022	0.000	0.000	0.001	0.002	−0.009	0.013	0.022	−0.035	0.078	0.002	0.000	0.005
Model 3	−0.030	−0.108	0.048	−0.045	−0.115	0.024	0.000	−0.001	0.001	0.002	−0.009	0.013	0.022	−0.035	0.078	0.002	0.000	0.005
Model 4	−0.038	−0.116	0.039	−0.051	−0.120	0.019	0.000	−0.001	0.001	0.003	−0.007	0.014	0.021	−0.035	0.078	0.001	−0.001	0.004
Wald‐statistic (model 1)	*p* = 0.74	*p* = 0.81	*p* = 0.01	*p* = 0.30	*p* = 0.65	*p* = 0.53

Model 1: Adjusted for offspring sex, height, and age at time of outcome measurement

Model 2: Maternal confounder-adjusted model: as model 1 plus adjustment for maternal age, ethnicity, pre-pregnancy BMI, nulliparity, social class, smoking, alcohol consumption

Model 3: Birth outcome-adjusted model: as model 2 plus adjustment for gestational age and birthweight

Model 4: Offspring adiposity-adjusted model: as model 3 plus adjustment for BMI child

* *p* < 0.05

The Wald-statistic tests the equality of the effects of depressive symptoms between week 18 and 32 in model 1.

Table 4 compares the multivariable associations of maternal depressive symptoms with offspring vascular outcomes to the equivalent associations of paternal symptoms. Paternal depressive symptoms or anxiety were not associated with any of the vascular outcomes in any models. For almost all associations, there was statistical evidence that the paternal–offspring associations were consistent with the maternal–offspring associations. There was some statistical evidence that the inverse association of maternal stress with offspring DBP was different to the paternal null association (Wald-statistic *p* = 0.04).

**Table 4.** Comparison of multivariable associations of maternal and paternal depressive symptoms (*n* = 3589) and anxiety (*n* = 3576) at partner's gestational week 18 with offspring vascular phenotypes at mean age 10.7 years

	Systolic blood pressure			Diastolic blood pressure			Flow-mediated dilatation			Pulse wave velocity			Distensibility coefficient			Brachial artery diameter		
	β	95% CI		β	95% CI		β	95% CI		β	95% CI		β	95% CI		β	95% CI	
		Lower	Upper		Lower	Upper		Lower	Upper		Lower	Upper		Lower	Upper		Lower	Upper
Paternal depressive symptoms
Model 1	−0.04	−0.12	0.04	−0.02	−0.08	0.05	0.000	−0.001	0.000	0.00	−0.01	0.01	0.01	−0.05	0.06	0.002	−0.001	0.004
Model 2	−0.04	−0.12	0.04	−0.02	−0.09	0.05	0.000	−0.001	0.000	−0.01	−0.02	0.01	0.01	−0.05	0.06	0.002	−0.001	0.004
Model 3	−0.04	−0.12	0.04	−0.02	−0.09	0.05	0.000	−0.001	0.000	−0.01	−0.02	0.01	0.01	−0.05	0.06	0.002	−0.001	0.004
Model 4	−0.04	−0.12	0.03	−0.02	−0.09	0.05	0.000	−0.001	0.000	0.00	−0.01	0.01	0.01	−0.05	0.06	0.001	−0.001	0.004
Maternal depressive symptoms
Model 1	−0.05	−0.11	0.02	−0.10*	−0.16	−0.04	0.000	−0.001	0.001	−0.01	−0.02	0.00	−0.01	−0.06	0.03	0.003*	0.001	0.005
Wald-statistic	*p* = 0.94	*p* = 0.04	*p* = 0.41	*p* = 0.70	*p* = 0.56	*p* = 0.23
Paternal anxiety
Model 1	−0.04	−0.15	0.07	−0.04	−0.13	0.06	0.000	−0.001	0.001	−0.01	−0.02	0.01	0.01	−0.07	0.08	0.002	−0.002	0.005
Model 2	−0.02	−0.12	0.09	−0.03	−0.12	0.07	0.000	−0.001	0.001	−0.01	−0.02	0.01	0.00	−0.08	0.08	0.002	−0.001	0.006
Model 3	−0.01	−0.12	0.10	−0.03	−0.12	0.07	0.000	−0.001	0.001	−0.01	−0.02	0.01	0.00	−0.08	0.08	0.002	−0.001	0.006
Model 4	−0.02	−0.13	0.09	−0.03	−0.13	0.07	0.000	0.000	0.000	−0.01	−0.02	0.01	0.00	−0.08	0.08	0.002	−0.002	0.005
Maternal anxiety
Model 1	−0.03	−0.12	0.06	−0.06	−0.13	0.02	0.000	−0.001	0.000	0.00	−0.02	0.01	0.02	−0.05	0.08	0.004*	0.001	0.007
Wald-statistic	*p* = 0.80	*p* = 0.98	*p* = 0.68	*p* = 0.88	*p* = 0.77	*p* = 0.18

Model 1: Adjusted for offspring sex, height, and age at time of outcome measurement

Model 2: Maternal confounder-adjusted model: as model 1 plus adjustment for maternal age, ethnicity, pre-pregnancy BMI, nulliparity, social class, smoking, alcohol consumption

Model 3: Birth outcome-adjusted model: as model 2 plus adjustment for gestational age and birthweight

Model 4: Offspring adiposity-adjusted model: as model 3 plus adjustment for BMI child

* *p* < 0.05

The Wald-statistic tests the equality of the effects of depressive symptoms between mother and father in model 1.

Table 5 shows the multivariable associations of postnatal maternal depressive symptoms and anxiety with offspring vascular function. Consistent with the findings of a lack of associations at each time point with offspring outcomes and of the strong correlations between symptoms at each time point, maternal postnatal symptoms were not strongly associated with overall adverse offspring vascular outcomes. There was some evidence that offspring of mothers with postnatal depressive symptoms or anxiety had a lower SBP and DBP. These associations were not explained by gestational duration, birthweight, or offspring age at vascular assessment.

**Table 5.** Multivariable associations of maternal postnatal depressive symptoms (*n* = 3421) and anxiety (*n* = 3375) with offspring vascular phenotypes at mean age 10.7 years

	Systolic blood pressure			Diastolic blood pressure			Flow-mediated dilatation			Pulse wave velocity			Distensibility coefficient			Brachial artery diameter		
	β	95% CI		β	95% CI		β	95% CI		β	95% CI		β	95% CI		β	95% CI	
		Lower	Upper		Lower	Upper		Lower	Upper		Lower	Upper		Lower	Upper		Lower	Upper
Depressive symptoms
Model 1	−0.072*	−0.134	−0.011	−0.080*	−0.135	−0.025	0.000	0.000	0.001	−0.002	−0.010	0.007	−0.006	−0.051	0.038	0.002	0.000	0.004
Model 2	−0.070*	−0.132	−0.008	−0.080*	−0.135	−0.025	0.000	0.000	0.001	−0.003	−0.011	0.006	−0.010	−0.055	0.035	0.001	−0.001	0.003
Model 3	−0.071*	−0.133	−0.009	−0.080*	−0.135	−0.025	0.000	0.000	0.001	−0.003	−0.011	0.006	−0.010	−0.055	0.034	0.001	−0.001	0.003
Model 4	−0.080*	−0.141	−0.018	−0.086*	−0.141	−0.031	0.000	0.000	0.001	−0.001	−0.010	0.007	−0.011	−0.056	0.034	0.001	−0.001	0.002
Anxiety
Model 1	−0.096*	−0.181	−0.011	−0.114*	−0.191	−0.038	0.001	−0.001	0.001	−0.004	−0.016	0.007	−0.001	−0.063	0.060	0.002	−0.001	0.005
Model 2	−0.084	−0.170	0.002	−0.109*	−0.186	−0.032	0.000	−0.001	0.001	−0.006	−0.018	0.006	−0.007	−0.070	0.055	0.002	−0.001	0.005
Model 3	−0.086	−0.171	0.000	−0.109*	−0.186	−0.033	0.000	−0.001	0.001	−0.006	−0.018	0.006	−0.007	−0.070	0.055	0.002	−0.001	0.005
Model 4	−0.094*	−0.179	−0.009	−0.115	−0.191	−0.038	0.000	−0.001	0.001	−0.005	−0.016	0.007	−0.008	−0.070	0.055	0.001	−0.002	0.004

Model 1: Adjusted for offspring sex, height, and age at time of outcome measurement

Model 2: Maternal confounder-adjusted model: as model 1 plus adjustment for maternal age, ethnicity, pre-pregnancy BMI, nulliparity, social class, smoking, alcohol consumption

Model 3: Birth outcome-adjusted model: as model 2 plus adjustment for gestational age and birthweight

Model 4: Offspring adiposity-adjusted model: as model 3 plus adjustment for BMI child

* *p* < 0.05.

## Discussion

We have found no convincing evidence that prenatal psychosocial stress, in the form of maternal depressive symptoms or anxiety, causes adverse levels of offspring blood pressure, endothelial function, arterial stiffness, brachial artery distensibility, or brachial diameter assessed at age 10–11 years via intrauterine mechanisms. Indeed for most outcomes, there was no association. Furthermore, the point estimates for the maternal–offspring association were very similar to those for the paternal–offspring associations, providing further evidence against the hypothesis that exposure to maternal stress *in utero* causes adverse developmental effects on vascular structure/function. Maternal depressive symptoms and anxiety in the postnatal period were associated with lower blood pressure in the offspring (i.e. in the opposite direction to what might be expected), though the magnitudes of association were small. As postnatal stress was not the prime focus of this paper, this could be a chance finding and requires further replication and detailed assessment to ascertain whether this is robust.

Our main findings seem to be in line with the only previous study of a somewhat similar association in a general population cohort that we are aware of, in which we did not find an association between prenatal maternal depressive symptoms and anxiety with offspring blood pressure in Dutch children at age 5–7.^21^ However, that previous study did report an association between the accumulation of multiple prenatal maternal stressors and higher offspring systolic and diastolic blood pressure. In the current study, we do not have a measure of the accumulation of multiple psychosocial stressors available. It is possible that the accumulation of different stressors can exert a programming effect on the offspring, which is not identified in a study such as this that assesses just two sources of stress (depression/anxiety) that are highly related to each other. Maternal cortisol early in pregnancy appears to be associated with both increased vascular resistance and lower arterial elasticity in children aged 5–7.^32,33^ Possibly, the stressors in the current study are not causing sufficient rises in maternal cortisol concentrations to exert effects on offspring arterial parameters. Of note the evidence for a direct association between psychosocial stress and cortisol, and thus the role of cortisol in fetal programming via psychosocial stress, has been a main point of discussion in a recent review by Glover et al.^6^ Nonetheless our results are important as they strongly suggest that levels of maternal stress in pregnancy that are experienced by a relatively large proportion of pregnant women are unlikely to adversely affect their offspring's vascular health.

It is possible that there are specific sensitive periods in which maternal stress has an effect on future cardiovascular function.^34^ However, the clear lack of associations at either 18 weeks or 32 weeks and the correlation between symptoms across these two time points and with the postnatal measurement (suggesting women would be ranked similarly throughout pregnancy) argues against this as an explanation for our null results.

The associations of maternal symptoms with wider brachial diameter and lower diastolic blood pressure were in the opposite direction to what we hypothesized and require further replication before one could consider these are not due to chance.

The large sample size is a strength of the current study, but as in most cohort studies, selective loss to follow up was present. Those who have remained in the cohort tend to be of higher socioeconomic position^24,25^ and we have shown that those who were included in the analyses here had lower parental depressive and anxiety scores than those who were excluded because of missing outcome or covariable data (see supplementary table, available online). If the association of parental symptoms with offspring outcomes was strong and inverse in those lost to follow up, then we may have biased null findings due to selection bias, but in separate studies exploring selection bias due to loss to follow up/missing data any bias tends to be small^35,36^ and we doubt this would be sufficient to produce important associations here.

Whilst the parental symptoms that we have assessed here use validated tools, they are not the same as clinical diagnoses and we cannot make any conclusions about the effects of more severe clinical depression or anxiety in terms of its association with offspring vascular function.

In conclusion, our findings suggest that maternal prenatal psychosocial stress does not cause adverse offspring vascular function in 10–11 year old children via intrauterine mechanisms. These results do not exclude the possibility that severe prenatal clinical depression or anxiety cause adverse offspring vascular function via intrauterine mechanisms, but to date we are not aware of any study that has explored that possibility in humans.

## Supplementary Material

Supplementary material
