# Supplementary material for: The association of maternal prenatal psychosocial stress with vascular function in the child at age 10–11 years: findings from the Avon longitudinal study of parents and children
Source: Eur J Prev Cardiol. 2013 Apr 4;21(9):1097–108. doi: 10.1177/2047487313486039 (PMC4230381; doi:10.1177/2047487313486039)
Supplement: Supplementary material [file CPR_486039_supplementary_data.doc]

Supplementary Table

*Characteristics compared between those who were included and excluded in the analyses*

|  | Included (*n*=4318) | Excluded (*n*=1301) | *p*-value |
| --- | --- | --- | --- |
| Maternal |  |  |  |
| Age at delivery (years) | 29.3±4.4 | 28.7±4.8 | *p*&#60;0.001 |
| White ethnicity (yes) | 98.7 | 98.0 |  |
| Pre-pregnancy body mass index (kg/m^2^) | 22.8±3.6 | 23.2±4.2 | *p*&#60;0.01 |
| Obesity (body mass index &#8805;30 kg/m^2^) (yes) | 4.5 | 3.6 | p=0.02 |
| Social class |  |  | *p*&#60;0.01 |
| I (professional/managerial) | 7.1 | 5.2 |  |
| II | 35.9 | 31.1 |  |
| III (non-manual) | 41.7 | 43.6 |  |
| III (manual) | 6.3 | 10.0 |  |
| IV | 7.6 | 8.7 |  |
| V (unskilled manual workers | 1.4 | 1.5 |  |
| Primiparous (yes) | 49.4 | 35.3 | *p*&#60;0.001 |
| Smoking |  |  | *p*&#60;0.001 |
| Non-smoking | 77.3 | 69.7 |  |
| Stopped before pregnancy or in first trimester | 11.1 | 12.1 |  |
| Smoked during pregnancy | 11.6 | 18.2 |  |
| Alcohol |  |  |  |
| None | 43.7 | 43.4 |  |
| &#60;1 glass/week | 41.0 | 40.5 |  |
| &#62;1 glass/week | 13.7 | 14.6 |  |
| &#62;1 glass/day | 1.5 | 1.6 |  |
| Pregnancy-related hypertension (yes) | 15.5 | 16.8 |  |
| Maternal stress |  |  |  |
| Anxiety at gestational week 18 | 5±3.4 | 5±3.4 | *p*&#60;0.001 |
| Anxiety at gestational week 32 | 5±3.4 | 5±3.6 | *p*&#60;0.001 |
| Depressive symptoms at gestational week 18 | 6±4.5 | 7±4.6 | *p*&#60;0.001 |
| Depressive symptoms at gestational week 32 | 6±4.8 | 7±5.1 | *p*&#60;0.001 |
| Pre- and postnatal anxiety categories |  |  | *p*&#60;0.001 |
| Low anxiety at all time points | 59.9 | 52.2 |  |
| Prenatal anxiety only | 19.8 | 2.5 |  |
| Postnatal anxiety only | 5.8 | 5.9 |  |
| High anxiety at all time points | 14.5 | 18.3 |  |
| Pre- and postnatal depressive symptom categories |  |  | *p*&#60;0.001 |
| Low depressive symptoms at all time points | 61.6 | 53.9 |  |
| Prenatal depressive symptoms only | 15.0 | 16.9 |  |
| Postnatal depressive symptoms only | 8.7 | 9.1 |  |
| High depressive symptoms at all time points | 14.6 | 20.1 |  |
| Child at birth |  |  |  |
| Born through caesarean section (yes) | 9.6 | 10.2 |  |
| Sex (boys) | 49.4 | 50.2 |  |
| Gestational age (weeks) | 39.6±1.6 | 39.5±1.9 |  |
| Premature (&#60;37 weeks) (yes) | 3.7 | 5.6 | *p*&#60;0.01 |
| Birthweight (g) | 3456±494 | 3440±570 |  |
| Child at age 10 assessment |  |  |  |
| Age (years) | 10.7±0.2 | 10.7±0.3 | *p*&#60;0.001 |
| Height (cm) | 144±7 | 144±7 |  |
| Body mass index (kg/m^2^) | 18.1±3.0 | 18.4±3.3 | *p*&#60;0.01 |
| Systolic blood pressure (mmHg) | 104±9 | 104±9 |  |
| Diastolic blood pressure (mmHg) | 60±8 | 60±8 |  |
| Flow-mediated dilatation (mm) | 0.21±0.08 | 0.21±0.09 |  |
| Pulse wave velocity (m/s) | 7.55±1.22 | 7.57±1.27 |  |
| Distensibility coefficient (% per mmHg) | 12.5 | 6.1 | 12.7 |
| Brachial artery diameter (mm) | 2.67±0.30 | 2.69±0.32 | *p*=0.04 |

Values are mean±SD or %.
